# Commentary: Clinical utility of anthropometric parameters in identifying glucose dysregulation in women with polycystic ovary syndrome

**DOI:** 10.3389/fendo.2026.1804024

**Published:** 2026-03-26

**Authors:** Jiewen Liu, Zhiwei Hu, Jun Pan

**Affiliations:** Department of Endocrinology, The First People’s Hospital of Jiashan, Jiashan Hospital Affiliated of Jiaxing University, Jiaxing, Zhejiang, China

**Keywords:** anthropometric indices, cardiometabolic health, insulin resistance, metabolic characteristics, polycystic ovary syndrome

## 1 Introduction

Nowak et al.’s study offers important evidence on non-invasive screening for glucose dysregulation in women with polycystic ovary syndrome (PCOS) using anthropometric indices (1). The identification of optimal cut-off points for indices such as visceral adiposity index (VAI) and body mass index (BMI) is clinically relevant, considering the high prevalence of insulin resistance (IR) among women with PCOS. However, two critical issues in study design and data analysis may limit the generalizability and reliability of the findings, and these issues require further clarification.

## 2 Subsections

### 2.1 Lack of Phenotype-Stratified Analysis Overlooks Metabolic Heterogeneity in PCOS

PCOS is a heterogeneous disorder with distinct phenotypes as defined by the Rotterdam criteria, including hyperandrogenism-predominant, ovulatory dysfunction-predominant, and combined phenotypes (2). These phenotypes exhibit significant differences in metabolic risk; compared with non-hyperandrogenic polycystic ovary syndrome, patients with hyperandrogenic polycystic ovary syndrome have a higher risk of insulin resistance, anovulation, metabolic disorders and elevated triglycerides (3). Nowak et al. did not stratify their analyses by PCOS phenotype, potentially conflating the predictive value of anthropometric indices across subgroups with varying metabolic profiles. This oversight may lead to suboptimal cut-off points that fail to capture IR risk in specific phenotypes, reducing the clinical utility of the proposed indices for personalized screening.

#### 2.1.1 Solution

All analyses were stratified by PCOS phenotype (hyperandrogenic, ovulatory dysfunction-predominant, combined) to assess phenotype-specific predictive performance of anthropometric indices. Separate cut-off points and diagnostic metrics (AUC, sensitivity, specificity) were reported for each subgroup. This approach aligns with recent high-impact studies, which emphasize that PCOS phenotypes have distinct metabolic risk trajectories (4, 5). Consequently, tailored risk assessment and management are required. For instance, hyperandrogenic phenotypes often need more frequent metabolic monitoring because of their strong association with severe insulin resistance (IR) (4). In contrast, ovulatory phenotypes may benefit from less intensive but targeted screening.

### 2.1.2 Inadequate adjustment for hyperandrogenism as a confounding factor biases predictive validity

Hyperandrogenism is a core diagnostic feature of PCOS and an independent driver of glucose dysregulation and IR, even after accounting for adiposity (4). It both directly impairs insulin signaling in hepatocytes and adipocytes, and indirectly exacerbates metabolic dysfunction by reducing sex hormone-binding globulin (SHBG) and altering lipid metabolism (6). Nowak et al.’s logistic regression models only included anthropometric indices as predictors and excluded adjustment for hyperandrogenic markers (such as serum testosterone, free androgen index [FAI], or clinical hirsutism). This omission introduces confounding bias: the observed strong associations (e.g., OR = 57.0 for BMI and VAI) may partially reflect synergistic effects of hyperandrogenism rather than the independent predictive power of adiposity itself. Without adjusting for this key confounder, the proposed cut-off points may overestimate the utility of anthropometric indices in hyperandrogenic PCOS and underestimate their utility in non-hyperandrogenic subgroups.

#### 2.1.3 Solution

First, incorporating the hyperandrogenic markers (the serum testosterone, FAI) as confounding variables in logistic regression models to isolate the independent association between anthropometric indices and glucose dysregulation. Second, performing stratified analyses by hyperandrogenism status (hyperandrogenic vs. non-hyperandrogenic) to compare the performance of indices across subgroups. This adjustment ensures that the study’s conclusions reflect the true predictive value of adiposity-related indices, independent of the direct metabolic effects of androgens.

## 3 Discussion

Nowak et al.’s study provides valuable non-invasive screening insights for PCOS-related glucose dysregulation, but phenotype stratification and hyperandrogenism adjustment are critical to strengthening the conclusions. Phenotype-specific analyses address PCOS metabolic heterogeneity; hyperandrogenic subtypes carry higher IR and metabolic risks, requiring tailored cut-off points for accurate screening. Adjusting for hyperandrogenism eliminates confounding bias, because it independently contributes to glucose dysregulation beyond adiposity, ensuring the observed associations reflect anthropometric indices’ true predictive power. These improvements would align the study with current clinical guidelines and facilitate the translation of its findings into routine clinical practice. The original study’s strengths in evaluating multiple anthropometric indices are significant, and addressing these issues would further elevate its clinical impact.
